# The Opium Treatment of Peritonitis

**Published:** 1891-10

**Authors:** 


### THE OPIUM TREATMENT OF PERITONITIS.

It gives us great pleasure to call the attention of the profession to the report of a case of peritonitis by Dr. Himmelsbach, that appears in this number of the Journal. It illustrates well the great superiority of the *true* Alonzo Clark morphine treatment of acute general peritonitis over all other forms of treatment.

In this case the salts treatment had been tried and had failed ; the small-dose morphine treatment had been tried with lamentable results, and when the child was brought to the hospital, her condition was exceedingly critical. *Morphine given for its effect, regardless of size of dose*, saved the child's life. Think of it ! One hundred and seventy grains of morphine given hypodermically within seventeen days to a child ten years of age, one *single* dose containing *five* grains, and several containing, each, *four* grains, and the respiration not falling below fifteen per minute ! Still there are a great many medical men who say, and apparently think, that there is no especially great tolerance for the drug in acute peritonitis, and they are afraid to save their patients' lives. Only ten days ago we actually heard a physician say that he had had a case of acute peritonitis in a young girl to whom he gave at one dose as much as three-quarters of a grain of morphine hypodermically, and he was afraid to go further, *preferring to have the patient die of peritonitis than of morphine.* The paitient died of course, and such a case would be reported as a failure of the morphine treatment instead of a failure of the judgment of the physician, as it should be.

"Give opium to within an inch of your patient's life," was the direction that Dr. Clark gave one of his assistants. A good rule to follow is to give the morphine in doses large enough to keep the patient absolutely free from pain and in a semi-narcotized condition, even if the respirations fall as low as ten or eight per minute in securing that condition. DeL. R.

